# Effects of hybrid emergency department on extracorporeal cardiopulmonary resuscitation in out-of-hospital cardiac arrest patients

**DOI:** 10.1016/j.resplu.2024.100764

**Published:** 2024-09-05

**Authors:** Takashi Nakata, Daisuke Kudo, Yasushi Kudo, Atsushi Tanikawa, Ken Katsuta, Hiroyuki Ohbe, Masakazu Kobayashi, Akira Suda, Satoshi Yasuda, Shigeki Kushimoto

**Affiliations:** a Department of Cardiovascular Medicine, Tohoku University Graduate School of Medicine, Sendai, Japan; b Division of Emergency and Critical Care Medicine, Tohoku University Graduate School of Medicine, Sendai, Japan

**Keywords:** Hybrid emergency department, Extracorporeal cardiopulmonary resuscitation, Out-of-hospital cardiac arrest

## Abstract

**Background:**

Hybrid emergency department (ED), which are equipped with fluoroscopy and computed tomography has been developed in Japan as a novel emergency care room. Although hybrid ED is effective in improving the outcomes of severe trauma, its influence on the management of out-of-hospital cardiac arrest (OHCA) requiring extracorporeal cardiopulmonary resuscitation (ECPR) remains unclear.

**Objectives:**

The aim of this study was to elucidate the impact of hybrid ED on ECPR procedures and outcome in OHCA patients focusing on time from hospital arrival to establishment of ECPR.

**Methods:**

A retrospective single-center cohort study was conducted, including adult OHCA patients who underwent ECPR between April 2013 and March 2022. Patients treated in conventional ED were compared with those in hybrid ED. Primary outcome was time from hospital arrival to ECPR initiation. Secondary outcomes included favorable neurological outcome at 30 days and incidence of cannulation-related adverse events.

**Results:**

Hybrid ED installation led to a significant decrease in time to ECPR initiation. In the interpreted time series analysis for the time from hospital arrival to establishment ECPR, there was statistically significant upward level change and downward trend change after the installation of hybrid ED. These results mean the time from hospital arrival to the establishment of ECPR was prolonged just after installation of hybrid ER, and the time from hospital arrival to the establishment of ECPR was shortened over time. There were no statistically significant differences between the conventional and hybrid ED groups on the favorable neurological outcome and cannulation-related adverse events.

**Conclusions:**

The installation of hybrid ED was associated with shortened time from hospital arrival to establishment of ECPR. Further evaluation is needed to elucidate the effects of hybrid ED on OHCA and determine an optimal strategy.

## Introduction

Out-of-hospital cardiac arrest (OHCA) is a major cause of death worldwide, with ∼350,000 cases annually in the United States.^1^ Despite advancements in pre-hospital care and cardiopulmonary resuscitation (CPR), the survival and neurological outcomes of patients who experience OHCA events remain poor.^2^

Extracorporeal membrane oxygenation (ECMO) during cardiopulmonary resuscitation is called extracorporeal cardiopulmonary resuscitation (ECPR), and some reports have suggested that it has beneficial effects on the outcomes of patients following OHCA events.^3^, ^4^ On the other hand, some have reported no difference in association to favorable neurological outcome between ECPR and conventional CPR.^5^, ^6^ In a systematic review and meta–analysis of randomized clinical trials of ECPR, ECPR was associated with a non-statistically significant higher rate of survival with a favorable neurological outcome.^7^ According to the Extracorporeal Life Support Organization (ELSO) guidelines, it is reasonable to consider ECPR for patients who do not respond to conventional CPR within 10–20 min.^8^ However, there are challenges and concerns associated with ECPR, including appropriate patient selection, complications, and costs. The hybrid emergency room (ER) is a novel type of resuscitation suite that was established in 2011 in Japan in an effort to address these challenges.

**Fig. 1:**
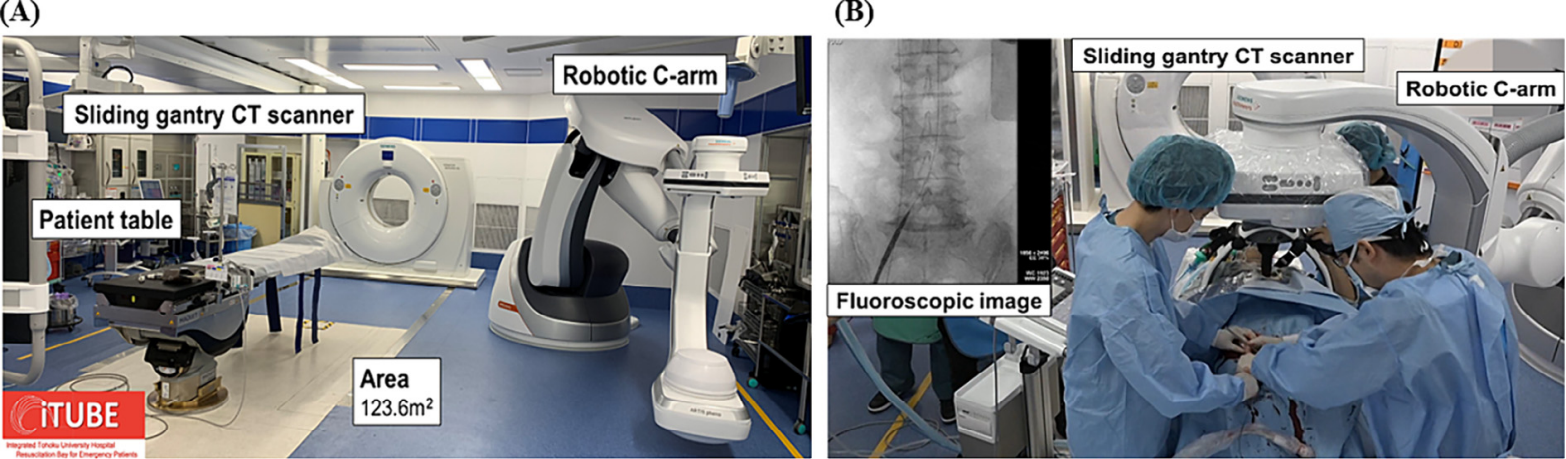
(A) Hybrid emergency room in Tohoku university hospital. (B) ECPR in hybrid emergency room.

A hybrid ER is an integrated ER equipped with computed tomography (CT), interventional radiology (IVR), and surgical operating suites. In the hybrid ER, resuscitation, CT imaging, endovascular intervention, and emergency surgery can be performed without patient transfer (Fig. 1A). The hybrid ER system is reported to be effective in reducing mortality in patients who have experienced severe trauma.^9^, ^10^ It has been hypothesized that patients with internal pathophysiological conditions may experience similar beneficial effects.^11^, ^12^

The hybrid ER enables the performance of ECPR with fluoroscopy without the need for a patient transfer to a separate angiography room (Fig. 1B). Thus, there is a possibility that a hybrid ER may reduce the complications associated with ECPR and shorten the time from calls to emergency medical services (EMS) to the initiation of ECPR, resulting in improved survival in cases of OHCA.^13^ However, the effect of hybrid ER use on ECPR procedures and outcomes remains unclear in patients who experience OHCA with cardiac causes requiring ECPR.

This study aimed to elucidate the impacts of hybrid ER use on ECPR procedures and outcomes in patients who experienced OHCA events that required ECPR, with a focus on the time from hospital arrival to the initiation of ECPR.

## Methods

### Study design and participants

This retrospective, observational, single-center study was conducted at Tohoku University Hospital. All of the protocols were approved by the Institutional Review Board of Tohoku University, Sendai, Japan (approval no.: 2023-1-742).

Adult patients with OHCA of presumed cardiac etiology who underwent ECPR between April 2013 and April 2022 were enrolled. The exclusion criteria were: age < 18 years old in accordance with the inclusion criteria of the SAVE-J II study, a registry of ECPR conducted in Japan^14^; non-cardiac cause of OHCA; body temperature < 30 °C at time of arrival to a hospital; patients who received ECPR in a conventional ER following the installation of the hybrid ER in the hospital; patients requiring ECPR for refractory ventricular fibrillation (VF) after arriving at the hospital.

The detail of the EMS system in our city is that under certain conditions, when an emergency call is received, an ambulance accompanied by a doctor, called a doctor car, goes to the scene, joins the emergency team, and transports the patient to the respective hospital. During the transport, intubation and administration of adrenaline and antiarrhythmic drug can be performed. In other cases, Emergency lifesaving technician equivalent to paramedics can perform indication-based defibrillation, use of mechanical chest compression device, a supraglottic airway device and administer adrenaline during transportation to the hospital. Our hospital installed hybrid ERs in May 2018, and we compared patients treated in the conventional ER (from April 2013 to April 2018) with those treated in the hybrid ER (from May 2018 to April 2022).

### ECPR procedure

From the pre-hospital patient information, ECPR was considered to have been performed in patients who had a good chance of survival following cardiac arrest—such as in cases where the OHCA event was witnessed, those who received high-quality CPR prior to arrival, and those in whom the initial cardiac rhythm was not asystole (based on the ELSO consensus statement).^8^ When ECPR was considered, the attending emergency physician asked the clinical engineers to prepare the ECMO circuit. Radiology technicians were asked to prepare portable X-ray radiography equipment in the conventional ER (in years preceding the installation of the hybrid ER), or the robotic C-arm in the hybrid ER. ECPR was performed by emergency physicians, using ultrasound-guided femoral vessel puncture in both the conventional and hybrid ERs. We typically selected the right femoral vein and artery as the cannulation sites. The positions of the guidewire and cannula were adjusted using a portable X-ray device in the conventional ER, and fluoroscopy-guided positioning in the hybrid ER. Chest compressions were continued during ECMO cannulation, using a mechanical chest compression device.

### Data collection

We analyzed data extracted from electronic and paper-based medical records. All patients were followed-up with until hospital discharge or death. The recorded patient characteristics are described in supplementary materials.

### Outcomes

The primary endpoint was the time from hospital arrival until the initiation of ECPR. Secondary endpoints were survival at 30 days after admission, neurological outcomes at 30 days after admission, cardiac recovery defined successful ECMO removal, estimated low-flow time, and incidence of cannulation-related adverse events. Neurological outcomes were evaluated using the cerebral performance category (CPC) scale. A CPC of 1 or 2 was defined as a favorable neurological outcome. Cannulation-related adverse events included cannula malpositioning and cannulation-related bleeding. Cannula malpositioning was defined as a case requiring adjustment of the cannula position or incorrect vessel cannulation, such as arterial-arterial or veno-venous cannulation. Cannulation-related bleeding included cannulation-site bleeding, retroperitoneal hemorrhage requiring blood transfusion, surgical intervention, or IVR.

### Statistical analysis

Continuous variables are shown as medians with their accompanying 25th and 75th percentiles, and categorical variables are shown as counts and percentages. A univariate analysis of the patients’ characteristics and outcomes was performed between patients treated in the conventional ER group and those in the hybrid ER one, using the Mann-Whitney *U* test for continuous variables and the Chi-squared test for categorical ones.

Changes in the primary outcome before and after the hybrid ER installation were evaluated using segmented linear regression with an interrupted time series,^15^ which is a method used in statistical and social science research to evaluate how a particular intervention or event affects change over time to evaluate the impact of installation of intervention. This method compares data before and after an intervention to identify its effects.

Data analysis involves pre- and post-intervention comparisons. Typically, we analyze in two ways

• **Change in level**: to see if the mean or level of the data changes immediately after the intervention.
• **Trend change:** to see if the trend of the data (increasing or decreasing trend) changes after the intervention compared to before the intervention.

In our subgroup analysis, we excluded patients who did not receive intensive care because of evident hypoxic encephalopathy on CT during the resuscitation phase, or other general conditions.

All statistical analyses were two-tailed, and differences with P values of < 0.05 were considered statistically significant. All analyses were performed using Stata/SE 17.0 (StataCorp, College Station, TX, USA).

## Results

### Participants

**Fig. 2:**
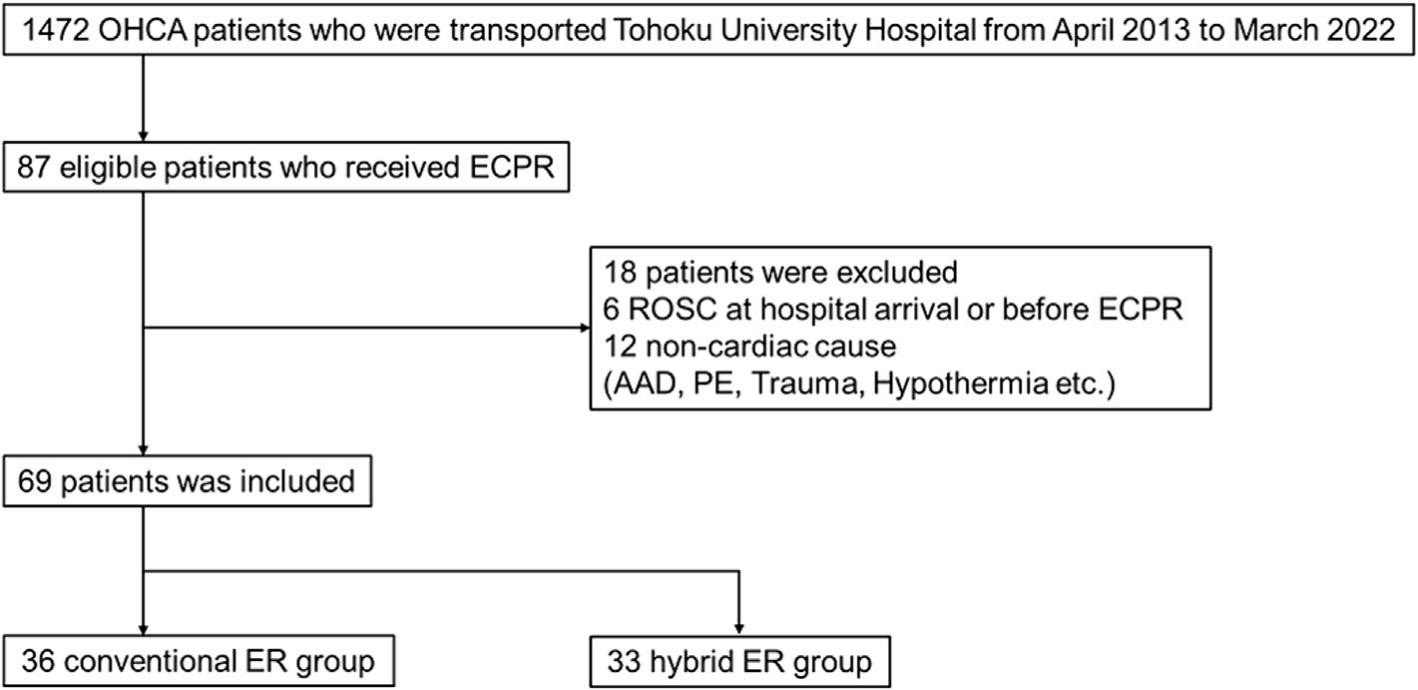
**Flowchart of the study patients**. OHCA, out of hospital cardiac arrest; ECPR, extracorporeal cardiopulmonary resuscitation; ROSC, return of spontaneous circulation; AAD, acute aortic dissection; PE, pulmonary embolism; ER, emergency room.

Over the study period, 1,472 patients who had experienced OHCA events were transported to Tohoku university hospital and 87 received ECPR. Of these, 18 were excluded (6 because of return of spontaneous circulation upon arrival to a hospital or before ECMO initiation, and 12 because of non-cardiac OHCA causes). As a result, 69 patients were included—36 patients in the conventional ER group, and 33 in the hybrid ER one (Fig. 2). Regarding baseline characteristics, the rate of bystander CPR and mechanical CPR during transport was higher in the hybrid ER group than in the conventional ER. The ratios of pre-existing comorbidities, ACS, place of cardiac arrest, initial rhythm, emergency CAG, PCI, and pre-hospital time course were comparable between the two groups (Table 1).

**Table 1:** Baseline characteristics in OHCA patients who received ECPR.

Variables	Conventional ER (*n* = 36)	Hybrid ER (*n* = 33)	P value
Age, years	56 (51–67)	52 (43–61)	0.06
Male	30 (83.3)	30 (90.1)	0.35
BMI, kg/m^2^	25.7 (22.5–28.0)	26.6 (24.4–29.3)	0.55
Comorbidities			
Hypertension	11 (30.1)	9 (27.3)	0.76
Hyperlipidemia	3 (8.3)	6 (18.2)	0.23
Diabetes mellites	8 (22.2)	6 (18.2)	0.68
Heart diseases	6 (16.7)	6 (18.2)	0.87
Witnessed arrest	24 (68.6)	25 (78.1)	0.38
Bystander CPR	15 (42.9)	22 (68.8)	0.03
Cause of arrest			
Acute coronary syndrome	17 (47.2)	11 (33.3)	0.24
Other cardiac	19 (52.8)	22 (66.7)	
Place			0.35
House	18 (50.0)	10 (30.3)	
Public space	8 (22.2)	13 (39.4)	
Workplace	5 (13.9)	5 (15.2)	
Ambulance	0 (0)	1 (3.0)	
Others	5 (13.5)	4 (11.8)	
Prehospital airway management			< 0.01
BVM	31 (86.1)	16 (48.5)	
SAD	2 (5.6)	13 (39.4)	
Intubation	3 (8.3)	4 (12.1)	
Prehospital adrenaline administration	12 (33.3)	15 (45.5)	0.31
Mechanical CPR	6 (16.7)	17 (51.5)	< 0.01
Initial cardiac rhythm on arrival			0.79
Ventricular fibrillation	26 (72.2)	27 (78.8)	
Pulseless electrical activity	5 (13.9)	4 (12.1)	
Asystole	5 (13.9)	3 (9.1)	
Shockable rhythm	26 (72.2)	26 (78.8)	0.53
Emergency coronary angiography	15 (41.6)	17 (51.5)	0.41
Percutaneous coronary intervention	10 (27.8)	9 (27.3)	0.96
Call to EMS arrival, minutes	7.5 (6.0–9.5)	7.0 (7.0–9.0)	0.43
Scene time, minutes	10 (7.0–12.0)	12 (9.0–14.0)	0.09
Transport time, minutes	9.0 (5.0–15.0)	8.0 (6.0–11.0)	0.77
Cardiac recovery	13 (36.1)	14 (42.4)	0.59

BMI, body mass index; CPR, cardiopulmonary resuscitation; EMS, emergency medical service; ROSC, return of spontaneous circulation; BVM, bag valve mask; SAD, Supraglottic airway device. Continuous variables are given as median (interquartile range, from 25th to 75th Percentiles). Categorical variables are given as count (percent).

BMI, body mass index; CPR, cardiopulmonary resuscitation; ROSC, return of spontaneous circulation; EMS, emergency medical services.

Continuous variables are given as median (interquartile range, from 25th to 75th Percentiles). Categorical variables are given as count (percent).

### Primary outcome—the change in ECPR time course before and after hybrid ER installation

**Fig. 3:**
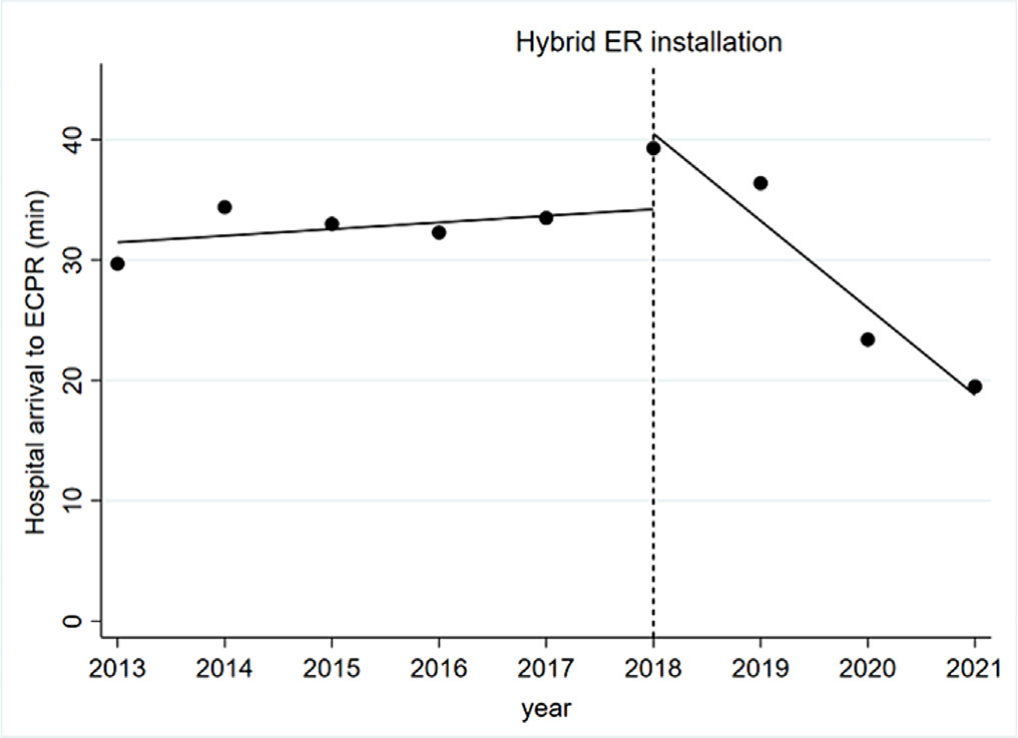
**Interrupted time-series analyses to evaluate the effect of hybrid ER installation on the time from hospital arrival to ECPR**. Each black point represents the mean time of hospital arrival to ECPR each fiscal year (from April to March), and the black lines represents the regression lines. ECPR, extracorporeal cardiopulmonary resuscitation; ER, emergency room.

Table 2 shows the time course of ECPR. The median time from hospital arrival to the establishment of ECPR was 35 (interquartile range, 24–44) min in the conventional ER group, and 24 (17–34) min in the hybrid ER group. Fig. 3 shows the mean time from hospital arrival until the establishment of ECPR in each fiscal year. In our interpreted time series analysis for the time from hospital arrival to the establishment of ECPR, there was a statistically significant upward level change (+6.3 [95% confidence interval, +2.1 to + 10.5] minutes; P=0.01) and downward trend change (–7.4 [–9.0 to –5.5]. min per year; P<0.01) after the installation of the hybrid ER. In our research, the time from hospital arrival to the establishment of ECPR was prolonged just after installation of hybrid ER. On the other hand, the time from hospital arrival to the establishment of ECPR was shortened over time, and that trend was not seen before hybrid ER installation.

**Table 2:** Time course of ECPR and results of interrupted time series analyses.

Time course (minutes)	Conventional ER (*n* = 36)	Hybrid ER (*n* = 33)	Level change, minutes, (95%CI)	P value	Trend change, minutes per year, (95%CI)	P value
Arrival to ECPR	35 (24–44)	24 (17–34)	6.3 (2.1, 10.5)	< 0.01	−7.4 (−9.0, −5.5)	0.010
Estimated low flow time	58 (49–75)	56 (46–70)	–	–	–	–

ER, emergency room; CI, confidence interval; IQR interquartile range; ECPR, extracorporeal cardiopulmonary resuscitation.

Continuous variables are given as median (interquartile range, from 25th to 75th percentiles).

### Secondary outcomes—neurological outcomes, cardiac recovery, estimated low-flow time, and cannulation-related adverse events

**Fig. 4:**
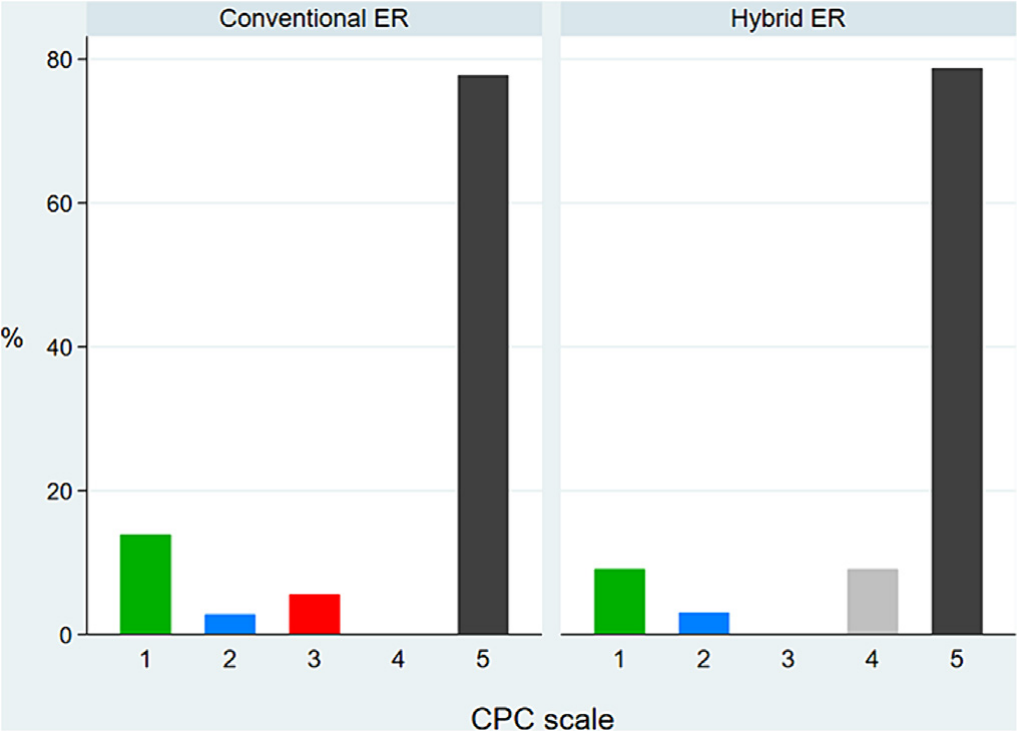
**Cerebral performance category at 30 days after admission.** A favourable neurologic outcome was defined as a Cerebral Performance Category (CPC) score of 1 or 2 (normal performance or mild disability with independence) on a scale of 1 to 5, with higher scores indicating more severe disability. ER, emergency room.

Fig. 4 shows the patients’ neurological outcomes at discharge. The ratio of favorable neurological outcomes, defined as a CPC of 1 or 2, did not differ significantly between the two groups (conventional ER group, 16.7% vs. hybrid ER group, 12.1%; P=0.48). There was no significant difference in the ratio of cardiac recovery and estimated low-flow time between the two groups (Table 1, Table 2). Table 3 shows all cannulation-related adverse events, which also did not differ significantly between the two groups (conventional ER group, 13.9% vs. hybrid ER group, 8.8%; P=0.53).

**Table 3:** ECPR related adverse events.

ECPR related adverse events (%)	Conventional ER (*n* = 36)	Hybrid ER (*n* = 33)	P value
Cannulation related bleeding	4 (11.1)	3 (9.1)	
Cannula malposition	1 (2.8)	0	
Total	5 (13.9)	3 (9.1)	0.53

ECPR, extracorporeal cardiopulmonary resuscitation; ER, emergency room.

Categorical variables are given as count (percent).

## Discussion

This study on patients who experienced OHCA events of cardiogenic causes showed that the installation of a hybrid ER was associated with a shorter time from hospital arrival until ECPR initiation. The effect of time reduction increased over time, as was demonstrated by the downward slope change after the installation of the hybrid ER in our interrupted time-series analysis.

### Effect of a hybrid ER on the establishment of ECPR and outcomes of patients following OHCAs

The time reduction in ECPR establishment cannot be attributed to a generalized effect of the hybrid ER, because various factors influence ECPR outcomes. However, the installation of a hybrid ER may reduce interfacility variability in procedures.^16^ Reduction of the time from hospital arrival to ECPR will not simply indicate the effectiveness of the hybrid ER as a suite, but rather the effectiveness of hybrid ER “system”—including preparation for patient acceptance from pre-hospital information, establishment of in-hospital systems such as the preparation of ECPR, and collaboration not only with physicians but other medical staff members as well—as was demonstrated by the slope change in our interrupted time series analysis. However, our interpreted time series analysis also showed a significant change in the time to ECPR establishment, with the time from hospital arrival to ECPR initiation being prolonged just after hybrid ER installation. Similarly, Kinoshita et al. reported that a beneficial effect on the survival of patients with severe trauma was only observed beginning two years after the installation of a hybrid ER.^9^

The installation of a hybrid ER shortened the time from hospital arrival to the establishment of ECPR, but the time is as twice as long as other prospective studies.^3^, ^5^ Basically, our department perform ECPR cannulation on right common femoral artery and vein by single cannulator with one or two assistants due to the availability of staff members, as ordinal emergency room treatment. If personnel availability allows, further time savings may be obtained by performing ECPR with the bifemoral approach. In addition, estimated low-flow time and neurological outcomes did not differ significantly between the two groups. Wengenmayer et al. reported that low-flow time correlated with survival and was an independent predictor of mortality in patients who received ECPR^17^ so the lack of difference in low-flow time may have resulted in no difference in neurological outcomes in this study. In our city, resuscitation for patients with cardiac arrest is performed to the extent that it does not delay hospital transport as a basic rule, and most patients experienced intra-arrest transport. As a result, the time of prehospital care is relatively short compared to previous report.^5^, ^6^ Since, intra-arrest transport to hospital compared with continued on-scene treatment was associated with lower probability of survival to hospital discharge,^18^ pre-hospital treatment system besides faster ECMO initiation may also be key to improving neurological outcomes in this patient demographic. In the SAVE-J2 study, a Japanese registry of patients who experienced cardiogenic OHCA events requiring ECPR, 4 of 36 participating centers had hybrid ERs, but their effects were not studied. The impact of hybrid ERs on ECPR in patients who experience OHCA is expected to become clearer with the future analysis of cases from multiple centers. To improve the outcomes of patients who experience OHCA events, hybrid ERs may play an important role in the chain of survival, helping to achieve a more immediate establishment of ECPR, treating the causes of cardiac arrest, and detecting ECPR complications earlier.

### Effect of hybrid ER on ECPR-associated complications

Fluoroscopically-assisted cannulation may represent a key advantage of the hybrid ER with regard to bleeding complications and cannula malpositioning; however, this study did not find a significant reduction in complications associated with cannulation. In this study, data from conventional ERs between 2013 and 2015 were recorded on paper medical records, and there were cases in which details of the procedure and CT images could not be obtained. Thus, complications may have been underestimated in the conventional ER group. Yang et al. suggested that an immediate whole-body CT scan following ECPR may represent a useful tool in patients who experience OHCAs when CPR-related injuries or non-cardiac causes of OHCA are suspected^19^ and that hybrid ERs may assist in making prompt diagnoses and treating the ECPR-related complications, as well as other treatable causes of OHCA. Additionally, the hybrid ER system facilitates post-ECPR procedures, including CAG and PCI, without patient transfers. The ECPR workflow may shorten door-to-balloon (DTB) time and improve the outcomes of patients with ST-segment elevation myocardial infarction (STEMI). Indeed, Mitsuhara et al. reported that the installation of a hybrid ER shortened the DTB time in patients with VF^20^; however, we did not perform CAG and PCI in the hybrid ER of our hospital during the observation period for this study due to the availability of cardiologist and medical staff of the catheter laboratory. Therefore, we could not evaluate the effects of the hybrid ER on CAG and PCI following ECPR.

### Study limitations

This study was subject to some limitations worth noting. This was a retrospective single-center cohort study, the patient population was not consistent between the two groups, and there were no concrete criteria for introducing ECPR. The number of patients was also relatively small; thus, we could not adjust for confounding factors or biases. Regarding the effect of reducing the time until ECPR initiation, it cannot be proven that there were no time-varying confounders such as increased personnel or changes in ECPR members; therefore, factors other than hybrid ER implementation are also possible. Finally, this study was a comparison conducted in a historical cohort, so the results may be affected by the proficiency of the staff members who performed the ECPR procedures, as well as whether the patients were transported to the hospital during the day or at night. Ethical considerations make randomized controlled trials on eligible patients difficult; therefore, future multicenter studies are warranted to fully elucidate the influence of hybrid ERs on patients who require ECPR following OHCA events.

## Conclusions

In this study regarding our initial experience with the practice, the time from hospital arrival to ECPR initiation was shortened, for patients who experienced OHCA events, following the installation of a hybrid ER at our hospital. However, improvements in their neurological outcomes were not observed. Future larger-scale multicenter prospective studies are warranted to fully elucidate the effects of hybrid ERs on ECPR.

## Funding source

This work was supported by JSPS KAKENHI, grant numbers 21K16074 and 23K15121.

## CRediT authorship contribution statement

**Takashi Nakata:** Writing – review & editing, Writing – original draft, Validation, Investigation, Formal analysis, Data curation. **Daisuke Kudo:** Writing – review & editing, Visualization, Supervision, Methodology, Conceptualization. **Yasushi Kudo:** Data curation. **Atsushi Tanikawa:** Data curation, Conceptualization. **Ken Katsuta:** Data curation, Conceptualization. **Hiroyuki Ohbe:** Writing – review & editing, Methodology, Formal analysis. **Masakazu Kobayashi:** Supervision. **Akira Suda:** Funding acquisition, Conceptualization. **Satoshi Yasuda:** Supervision. **Shigeki Kushimoto:** Writing – review & editing, Supervision.

## Declaration of competing interest

The authors declare that they have no known competing financial interests or personal relationships that could have appeared to influence the work reported in this paper.

## Figures and Tables

**Supplementary Fig. 1:**
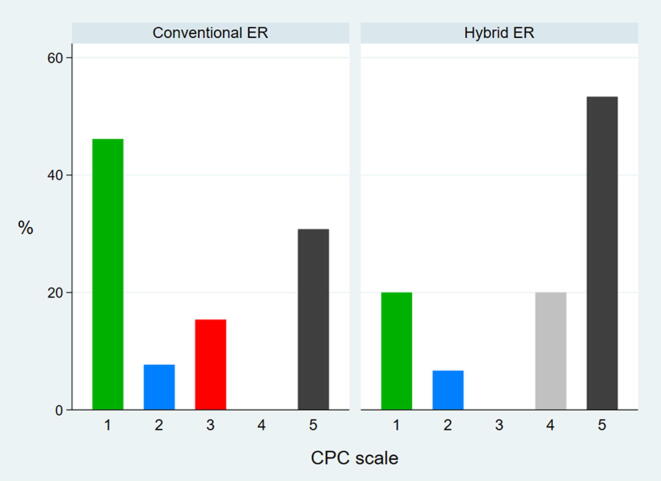
**Cerebral performance category at 30 days after admission in the subgroup analysis**. A favorable neurologic outcome was defined as a Cerebral Performance Category (CPC) score of 1 or 2 (normal performance or mild disability with independence) on a scale of 1 to 5, with higher scores indicating more severe disability, ER, emergency room.
