# Supplementary material for: Effects of hybrid emergency department on extracorporeal cardiopulmonary resuscitation in out-of-hospital cardiac arrest patients
Source: Resusc Plus. 2024 Sep 5;20:100764. doi: 10.1016/j.resplu.2024.100764 (PMC11408869; doi:10.1016/j.resplu.2024.100764)
Supplement: Supplementary Data 1 [file mmc1.docx]

**Supplementary materials**

**Abbreviations and acronyms:**

BMI = body mass index

CPR = cardiopulmonary resuscitation

CAG = coronary angiography

PCI = percutaneous coronary intervention

ACS = acute coronary syndrome

ECPR = extracorporeal cardiopulmonary resuscitation

EMS = emergency medical service

ER = emergency room

ED = emergency department

**1. Methods**

**1.1. Data collection**

We analyzed data extracted from electronic and paper-based medical records. All patients were followed-up with until hospital discharge or death. The recorded patient characteristics included age, sex, body mass index (BMI), comorbidities, arterial blood gas test results, presence of witnessed cardiac arrest and bystander CPR, prehospital airway management, prehospital adrenaline administration, mechanical CPR, initial cardiac rhythm at the scene and upon hospital arrival, and coronary angiography (CAG) and percutaneous coronary intervention (PCI). The causes of cardiac arrest were categorized into acute coronary syndrome (ACS), other cardiac causes (i.e., myopathy and myocarditis), and non-cardiac causes (e.g., aortic dissection, pulmonary embolism, hypothermia, and intracranial hemorrhage). We also recorded the time course of ECPR, including time from call to EMS until their arrival, transport time, scene time, time from arrival to hospital until ECPR initiation, and estimated low-flow time. The estimated low-flow time was defined as the time from cardiac arrest to the establishment of ECPR if the location of cardiac arrest was an ambulance and the time from calling EMS until the initiation of ECPR, if the location of cardiac arrest was outside of an ambulance.^1^ Since COronaVirus Infectious Disease (COVID) could have affected patient transport and acceptance, the time from call to EMS arrival and scene time in pre COVID (April 2013 to December 2019) and during post COVID (January 2020 to April 2022) was also recorded.

**2. Results**

**2.1. Participants**

In hybrid ER group, the ratio of mechanical CPR during transport was significant increased than the conventional ER group. The prehospital time course was comparable between pre COVID and during COVID (Table S1). In the blood test results, hemoglobin, chlorine, and c-reactive protein level was higher in the hybrid ER group than in the conventional ER (Table S2).

**2.2 Subgroup analysis**

Patients who did not receive intensive care were excluded from our subgroup analysis. Baseline characteristics, time from hospital arrival until ECPR initiation, and estimated low-flow time were comparable between the two groups (Tables S3 and S4). No significant difference was found in terms of the ratio of favorable neurological outcomes between the two groups (conventional ER group, 53.8% vs. hybrid ER group, 26.7%; P = 0.14; Figure S1). The patients who didn’t receive intensive care, ECMO was withdrawn in the ED, and other patients were admitted for palliative care. No patients were admitted for organ donation because the concrete criteria of organ donation in patients with ECMO was not defined in Japan during the observation period.

3. Refferences

1. Guglin M, Zucker MJ, Bazan VM, et al. Venoarterial ECMO for Adults: JACC Scientific Expert Panel. *J Am Coll Cardiol*. 2019;73:698-716. doi: 10.1016/j.jacc.2018.11.038

**4. Tables**

**Table S1. Prehospital time course pre and during COVID pandemic.**

| Variables | Pre COVID (n=42) | During COVID (n=27) | P value |
| --- | --- | --- | --- |
| Call to EMS arrival, minutes | 7.0 (6.0–9.0) | 8.0 (7.0–10.0) | 0.07 |
| Scene time, minutes | 10 (7.0–12.5) | 12 (9.0–14.0) | 0.09 |

COVID, COronaVirus Infectious Disease; EMS, emergency medical service

Continuous variables are given as median (interquartile range, from 25th to 75th percentiles).

**Table S2. Blood test results at the time of hospital arrival.**

| Variables | Conventional ER (n=36) | Hybrid ER  (n=33) | P value |
| --- | --- | --- | --- |
| pH | 6.97 (6.9–7.0) | 6.94 (6.8–7.1) | 0.51 |
| pCO_2_ (mmHg) | 73.8 (59.2–91.0) | 66.9 (45.3–102.0) | 0.71 |
| pO_2_ (mmHg) | 43.6 (15.7–97.0) | 56.3 (23.3–305.0) | 0.07 |
| BE mEq/L | –16.5 (–21.6– –14.6) | –16.6 (–25.3– –12.4) | 0.57 |
| HCO_3_ (mmol/L) | 15.7 (13.3–17.6) | 15.1 (11.9–19.4) | 0.75 |
| Lactate (mmol/L) | 11.0 (9.5–14.7) | 14.5 (9.9–17.0) | 0.06 |
| Na (mEq/L) | 142 (138–146) | 142 (140–146) | 0.53 |
| K (mEq/L) | 4.4 (3.6–4.9) | 4.1 (3.4–5.2) | 0.92 |
| Cl (mEq/L) | 110 (106–113) | 103 (101–105) | < 0.01 |
| Glucose (mEq/L) | 280.5 (168–340) | 306 (261–380) | 0.04 |
| Hb (g/dL) | 12.5 (10.6–15.0) | 14.6 (13.1–15.8) | 0.03 |
| WBC (10^3^/μL) | 9.7 (8.2–12.8) | 10.3 (7.5–14.7) | 0.55 |
| Plt (10^3^/μL) | 134 (76–212) | 180 (128–230) | 0.09 |
| T-bil (mg/dL) | 0.5 (0.4–0.8) | 0.5 (0.4–0.7) | 0.98 |
| Cre (mg/dL) | 1.17 (0.91–1.41) | 1.2 (1.0–1.4) | 0.31 |
| CRP (mg/dL) | 0.1 (0.1–0.3) | 0.07 (0.03–0.16) | 0.02 |
| Fibrinogen (mg/dL) | 220 (189–271) | 243.5 (180–294) | 0.50 |
| APTT | 32.3 (29.8–48.3) | 35.7 (30.8–52.3) | 0.55 |
| D-dimer (μg/mL) | 4.5 (3.2–9.3) | 9.4 (3.9–34.3) | 0.32 |

BE, base excess; Hb, Hemoglobin; WBC, white blood cell; Plt, platelet; T-bil, total bilirubin; Cre, creatinine; CRP, c-reactive protein; APTT, activated partial thromboplastin time.

Continuous variables are given as median (interquartile range, from 25th to 75th percentiles).

**Table S3. Baseline characteristics in OHCA patients who received intensive care after ECPR.**

| Variables | Conventional ER  (n=13) | Hybrid ER  (n=15) | P value |
| --- | --- | --- | --- |
| Age, years | 60 (54–65) | 53 (43–61) | 0.21 |
| Male sex | 11 (84.6) | 14 (93.3) | 0.46 |
| BMI, kg/m^2^ | 23.2 (21.1–27.5) | 26.8 (22.3–28.4) | 0.17 |
| Witnessed arrest | 10 (76.9) | 12 (80.0) | 0.84 |
| By stander CPR | 6 (46.2) | 9 (60.0) | 0.46 |
| Cause of arrest |  |  |  |
| Acute coronary syndrome | 5 (38.5) | 8 (53.3) | 0.43 |
| Other cardiac | 8 (61.5) | 7 (46.7) |  |
| Place |  |  | 0.66 |
| House | 5 (38.5) | 4 (26.7) |  |
| Public space | 5 (38.5) | 6 (40.0) |  |
| Workplace | 1 (7.7) | 3 (20.0) |  |
| Ambulance | 0 (0) | 1 (6.7) |  |
| Other | 2 (15.4) | 1 (6.7) |  |
| Initial cardiac rhythm on arrival |  |  | 0.36 |
| VF | 12 (92.3) | 14 (93.3) |  |
| PEA | 0 (0) | 1 (6.7) |  |
| Asystole | 1 (7.7) | 0 (0) |  |
| Emergency coronary angiography | 9 (69.2) | 11 (73.3) | 0.81 |
| Percutaneous coronary intervention | 5 (38.5) | 7 (46.7) | 0.66 |

BMI, body mass index; CPR, cardiopulmonary resuscitation; ROSC, return of spontaneous circulation.

Continuous variables are given as median (interquartile range, from 25th to 75th

Percentiles). Categorical variables are given as count (percent)

**Table S4. Time course of ECPR in OHCA patients who received intensive care after ECPR.**

| Time course  (minutes) | Conventional ER  (n=13) | Hybrid ER  (n=15) | P value |
| --- | --- | --- | --- |
| Arrival to ECPR | 25 (23–38) | 20 (14–33) | 0.11 |
| Estimated low flow time | 53 (47–71) | 43 (50–61) | 0.41 |

ER, emergency room; EMS, emergency medical service; ECPR, extracorporeal cardiopulmonary resuscitation.

Continuous variables are given as median (interquartile range, from 25th to 75th percentiles).
